# The pivotal role of immune functional assays in deciphering immune function alterations

**DOI:** 10.1093/cei/uxaf051

**Published:** 2025-08-09

**Authors:** Marion Debombourg, Guy Oriol, Caroline Dupre, Chloé Albert-Vega, Fabienne Venet, Thomas Rimmelé, REALISM Study Group, Anne Conrad, Florence Ader, Vincent Alcazer, VaccHemInf Study Group, Karen Brengel-Pesce, Aurore Fleurie, Sophie Trouillet-Assant, William Mouton

**Affiliations:** Joint Research Unit Hospices Civils de Lyon-bioMérieux, Hospices Civils de Lyon, Lyon Sud Hospital, Oullins-Pierre-Bénite, France; International Centre for Research in Infectiology (CIRI), INSERM U1111, CNRS UMR5308, ENS Lyon, Claude Bernard Lyon 1 University, Lyon, France; Joint Research Unit Hospices Civils de Lyon-bioMérieux, Hospices Civils de Lyon, Lyon Sud Hospital, Oullins-Pierre-Bénite, France; Joint Research Unit Hospices Civils de Lyon-bioMérieux, Hospices Civils de Lyon, Lyon Sud Hospital, Oullins-Pierre-Bénite, France; Joint Research Unit Hospices Civils de Lyon-bioMérieux, Hospices Civils de Lyon, Lyon Sud Hospital, Oullins-Pierre-Bénite, France; International Centre for Research in Infectiology (CIRI), INSERM U1111, CNRS UMR5308, ENS Lyon, Claude Bernard Lyon 1 University, Lyon, France; Immunology Laboratory, Hôpital Edouard Herriot, Hospices Civils de Lyon, Lyon, France; EA 7426 Pathophysiology of Injury‑Induced Immunosuppression, PI3, Claude Bernard Lyon 1 University‑bioMérieux‑Hospices Civils de Lyon, Hôpital Edouard Herriot, Lyon, France; Anesthesia and Critical Care Medicine Department, Hôpital Edouard Herriot, Hospices Civils de Lyon, Lyon, France; International Centre for Research in Infectiology (CIRI), INSERM U1111, CNRS UMR5308, ENS Lyon, Claude Bernard Lyon 1 University, Lyon, France; Infectious and Tropical Diseases Department, Hospices Civils de Lyon, Croix-Rousse Hospital, Lyon, France; Clinical Hematology Department, Hospices Civils de Lyon, Lyon Sud Hospital, Oullins-Pierre-Bénite, France; International Centre for Research in Infectiology (CIRI), INSERM U1111, CNRS UMR5308, ENS Lyon, Claude Bernard Lyon 1 University, Lyon, France; Infectious and Tropical Diseases Department, Hospices Civils de Lyon, Croix-Rousse Hospital, Lyon, France; Claude Bernard Lyon I University, Villeurbanne, France; International Centre for Research in Infectiology (CIRI), INSERM U1111, CNRS UMR5308, ENS Lyon, Claude Bernard Lyon 1 University, Lyon, France; Clinical Hematology Department, Hospices Civils de Lyon, Lyon Sud Hospital, Oullins-Pierre-Bénite, France; Joint Research Unit Hospices Civils de Lyon-bioMérieux, Hospices Civils de Lyon, Lyon Sud Hospital, Oullins-Pierre-Bénite, France; Joint Research Unit Hospices Civils de Lyon-bioMérieux, Hospices Civils de Lyon, Lyon Sud Hospital, Oullins-Pierre-Bénite, France; Joint Research Unit Hospices Civils de Lyon-bioMérieux, Hospices Civils de Lyon, Lyon Sud Hospital, Oullins-Pierre-Bénite, France; International Centre for Research in Infectiology (CIRI), INSERM U1111, CNRS UMR5308, ENS Lyon, Claude Bernard Lyon 1 University, Lyon, France; Joint Research Unit Hospices Civils de Lyon-bioMérieux, Hospices Civils de Lyon, Lyon Sud Hospital, Oullins-Pierre-Bénite, France; International Centre for Research in Infectiology (CIRI), INSERM U1111, CNRS UMR5308, ENS Lyon, Claude Bernard Lyon 1 University, Lyon, France

**Keywords:** immune dysfunction, immune functional assay, immune monitoring tool, transcriptomic analysis, method comparison

## Abstract

Growing evidence suggests that conventional immunomonitoring alone may not be sufficient to fully capture the complexity of immune dysfunctions. Immune functional assays (IFAs) have therefore emerged as valuable complementary tools, offering functional insights that extend beyond traditional phenotypic or quantitative approaches. Nevertheless, although *in vitro* stimulation represents a central component of IFAs, its specific contribution has never been rigorously evaluated, raising the critical question of whether this step is truly essential for detecting clinically relevant immune dysfunctions. To address this question, the present study compared gene expression levels (Nanostring) obtained from samples stimulated (TruCulture) or unstimulated (PaxGene) using the same analytical pipeline, in two distinct clinical settings: immune reconstitution following allogeneic hematopoietic stem cell transplantation (allo-HSCT) and sepsis progression. In allo-HSCT patients, post-stimulation data revealed immune heterogeneity and alterations related to ongoing immunosuppressive treatment or infectious event, not detected using unstimulated transcriptomic or cellular profiles alone. Similarly, post-stimulation transcriptomic profiles in patients with sepsis revealed immune clusters linked to disease severity and outcomes, surpassing traditional markers like mHLA-DR, while analyses from the unstimulated datasets failed to generate clinically relevant stratification. These findings emphasize the value of IFAs in uncovering immune function alterations that unstimulated assessments may miss, which could offer deeper insights into immune dysfunction. This study supports the use of IFAs as complementary tools to current clinical practices to enhance patient management by offering a functional view of immune system dynamics.

## Introduction

Over the past decade, the assessment of immune function has become a relevant approach for the evaluation of host immune capacities [1–3] and immune reconstitution quality [4, 5], as well as disease monitoring [1, 6, 7], across various research domains and clinical contexts. In this regard, Immune Functional Assays (IFAs) have emerged as essential tools [1, 8], the use of which requires an *in vitro* stimulation step followed by an analysis of the immune response induced by the stimulation, using a varying degree of analytical complexity. These analyses can range from single cytokine secretion measurement, such as interferon-gamma (IFN-γ), to more intricate phenotyping techniques, such as multiplex OMIC approaches [9]. A widely used IFA is the Interferon Gamma Release Assay (IGRA), commonly applied in infectious disease settings, such as for the diagnosis of *Mycobacterium tuberculosis* infection [10, 11] and more recently during the COVID-19 pandemic [12, 13]. Several studies have highlighted the advantages of employing IFA over traditionally used immunomonitoring tools, such as peripheral white blood cell counts or immune cell phenotyping [14–16]. More specifically, the employment of IFA, particularly TruCulture tubes (Myriad RBM, Austin, USA), combined with Nanostring technology to quantify mRNA gene expression level, allowed to obtain valuable insights into two distinct immunocompromised populations. In the field of allogenic hematopoietic stem cell transplantation (allo-HSCT), gene expression observed post-staphylococcal enterotoxin B (SEB) and post-lipopolysaccharide (LPS) stimulation allowed to identify immune function alterations associated with ongoing immunosuppressive treatment or infectious events at 6 months post-transplantation, which could not be revealed solely through cell count analyses, even though they are considered as reference markers for immune reconstitution [4]. In the setting of sepsis, transcriptomic data post-SEB stimulation in TruCulture tubes enabled to stratify a sepsis population according to severity and proved to be more effective than measurements of mHLA-DR, a marker classically used for immune monitoring [17].

However, while several studies have shown that IFAs can provide informative and complementary insights to routine tools in various infectious contexts (such as tuberculosis, sepsis, and others [1, 18–20]), none has performed statistical analyses to clearly demonstrate that the stimulation step involved in the IFA is essential to obtain this added value. In other terms, is the stimulation step truly necessary, and does it uncover immune dysfunctions that would otherwise remain undetected? Indeed, although studies using IFAs usually include an unstimulated control condition to normalize the results or validate the technique [21, 22], no direct comparison between stimulated and unstimulated conditions, using identical analytical pipelines, has been performed.

The present study thus aimed to evaluate the potential added value of performing an *in vitro* stimulation step for revealing immune function alterations by comparing gene expression levels obtained with and without stimulation in the two previously mentioned clinical settings: immune reconstitution following allo-HSCT and sepsis progression.

## Materials and methods

### Study design and population

In order to evaluate the added value of the *in vitro* stimulation step for revealing immune function alterations, gene analyses following the same analytical pipeline were performed on datasets obtained from stimulated and unstimulated samples from two different cohorts (allo-HSCT and sepsis). While the unstimulated datasets were generated specifically for the present study, the datasets of the stimulated samples were obtained from previously published studies and re-analyzed as new restricted datasets in order to allow direct comparisons with the unstimulated datasets. The classical immunomarkers used to monitor immune reconstitution were also obtained from the previous studies and re-analyzed on the present restricted datasets.

### Allo-HSCT cohort

Allo-HSCT recipients were included in the prospective, single-center cohort study ‘VaccHemInf’ (NCT03659773) between May 2018 and August 2020 at a median time of 6 months post-transplantation at the hematology department of the Lyon University Hospital (*Hospices Civils de Lyon* [HCL], Lyon, France). The study protocol was approved by the regional ethics committee (*Comité de Protection des Personnes Sud-Est V*, Grenoble, France, number 69HCL17_0769).

### Sepsis cohort

Patients with sepsis were included in the REALISM study (NCT02638779), a prospective longitudinal, single-center observational study, conducted in the anesthesiology and intensive care department of the Edouard Herriot hospital between December 2015 and June 2018 (HCL, France). Blood sampling was performed 3–4 days after septic shock onset. The study protocol was approved by the regional ethics committee (*Comité de Protection des Personnes Sud-Est II*, number 2015–42-2).

For each study, blood samples from age- and sex-matched healthy volunteers (HVs), collected and analyzed within the same time frame, were obtained from the national blood service (*Etablissement Français du Sang*), in accordance with regulatory authorizations for sample handling and storage, as approved by the regional ethics committee (*Comité de Protection des Personnes Sud-Est II*) and the French Ministry of Higher Education, Research and Innovation (*Ministère de lʼEnseignement supérieur, de la Recherche et de lʼInnovation, DC-2008–64*). Written informed consent was obtained from each healthy donor and from the patients or their relatives upon inclusion in these studies.

### Sample collection and RNA extraction

For each study, at the time of sampling, heparinized-whole blood was collected and incubated in TruCulture tubes (Myriad Rbm, Austin, TX) prefilled with either LPS or SEB, as previously described [4, 17]. Unstimulated whole-blood samples were also collected in PaxGene Blood RNA tubes (PreAnalytiX/QIAGEN Inc., Valencia, CA) and stored at −80°C. RNA extraction from TruCulture tubes, as well as gene analysis using Nanostring Technology had been performed in the original studies [4, 17]. Total RNA from whole-blood PaxGene samples was extracted using the PaxGene Blood RNA Kit (PreAnalytiX/QIAGEN Inc., Valencia, CA, USA) following the manufacturer’s instructions (see Supplementary Material and Methods). RNA concentration was estimated using the Nanodrop One spectrophotometer (Thermo Scientific, Swedesboro, NJ) according to the manufacturer’s instructions.

### Gene expression analysis

For both TruCulture and PaxGene samples, gene expression was evaluated using the NanoString technology through a 144- [4] and 89-gene panel [17] for the allo-HSCT and sepsis studies, respectively. Briefly, 300 ng of RNA were hybridized to the probes at 67°C for 18 hours using a thermocycler (Biometra, Tprofesssional TRIO, Analytik Jena AG, Jena, Germany). After removal of excessive probes, samples were loaded into the nCounter Prep Station (NanoString Technologies, Seattle, WA, USA) for purification and immobilization onto the internal surface of a sample cartridge for 2–3 hours. The sample cartridge was then transferred and imaged on the nCounter Digital Analyzer (NanoString Technologies), where color codes were counted and tabulated for each panel of genes. Data treatment and normalization were performed on nSolver analysis software (version 4.0, NanoString Technology) using internal controls and three housekeeping genes (detailed in Tables S1 and S2). Only genes with a normalized expression level exceeding the background noise threshold in more than 75% of individuals were considered for analysis.

### Clinical outcomes considered for the comparison between stimulated and unstimulated datasets

In order to compare the stimulated (TruCulture) and unstimulated (PaxGene) datasets in the allo-HSCT population, the two clinical outcomes initially studied were also considered herein: ongoing Herpesviridae infectious episode and ongoing immunosuppressive treatment. For the sepsis population, the clinical outcomes of the patients 28 days after inclusion used in the initial study (patients with sepsis, patients with sepsis who developed a hospital-acquired infection [HAI], and non-survivors) were also considered herein for the comparison of the stimulated and unstimulated datasets.

### Statistical analysis

The distribution of quantitative data was expressed as mean (range) or median (interquartile range, [IQR]) where appropriate. Normality testing was performed using the Shapiro–Wilk normality test. For the allo-HSCT population, the genes that were commonly expressed in both the stimulated (LPS and SEB) and unstimulated datasets were first identified. Then, volcano plots were drawn to visualize differentially expressed genes (DEGs) according to the clinical outcomes described above. To do so, Log_2_ transformed fold change (FC) between patients with or without the clinical outcome and Log_10_ transformed *P*-value obtained using the Mann–Whitney test or Student *t*-test, as appropriate, were calculated. DEGs were determined using the following limits: FC >2 or <−2 and a *P*-value of <0.05. The number of DEGs was then compared between the stimulated and unstimulated datasets for each clinical outcome. Additionally, receiver-operating characteristic curves (ROC) and the area under the curve (AUC) with its associated 95% confidence interval [95% CI] were built to identify the genes with the best performance for discriminating between the clinical outcomes. Finally, principal component analysis (PCA) was carried out using Partek Genomics Suite software (version 7.0; Partek Inc., St. Louis, MO). Euclidean distances from the centroid of the HVs for each allo-HSCT patient in both the stimulated and unstimulated datasets were then calculated, as previously described by Mouton et *al*. [4] Differences in standard deviations (SD) were calculated using paired Pitman–Morgan test. Differences in Euclidean distances according to the clinical outcomes were calculated using a non-parametric unpaired Wilcoxon test with Benjamini correction.

For the sepsis population, the genes commonly expressed in both the stimulated (SEB only) and unstimulated datasets were identified. Cluster analyses were then performed using the PAM method with correlation and average distance for each dataset, as previously described by Albert-Vega et *al.* [17]. Clinical and biological parameters were then compared between the different clusters obtained using either the stimulated or unstimulated dataset in order to evaluate whether these parameters could predict the distribution of patients within these clusters. Categorical variables were analyzed using the chi-squared test, and numerical variables using the *t*-test or Wilcoxon test, as appropriate. The alluvial plot was obtained via http://www.bioinformatics.com.cn, a free online platform for data analysis and visualization. Statistical analyses were conducted using GraphPad Prism software (version 10.4.1; GraphPad software, La Jolla, CA, USA), StatAid (version 1.3.1) [23] and R (version R.2.2). *P* values and adjusted *P* values (*P* adj) <0.05 were considered significant.

## Results

### Immune function assessment, a valuable tool to uncover immune function alterations in the context of allo-HSCT

We first aimed to evaluate the added value of the stimulation step in revealing immune function alterations during immune reconstitution after allo-HSCT. To that end, 59 of the 60 allo-HSCT recipients and 5 of the 10 HVs initially included in the study of Mouton et *al*. [4] were analyzed, as one patient sample did not pass quality control, and no PaxGene samples were available for 5 HVs. Allo-HSCT recipients and HVs were matched for age (median [IQR]: 44 [33–61] years for allo-HSCT recipients vs. 53 [49–61] years for HVs, *P-*value = 0.21) and sex (sex-male ratio 1.4 for allo-HSCT recipients vs. 1.5 for HVs, *P*-value >0.99). Regarding the hematological- and transplant-related characteristics of allo-HSCT recipients included at a median of 6.5 [5.8–8.3] months after transplantation, 52.5% had been transplanted due to acute myeloid leukemia, 35.6% had active graft-versus-host disease (GvHD) at inclusion, 32.2% were undergoing immunosuppressive treatment at inclusion, and 32.2% had a Herpesviridae infectious episode within two weeks of inclusion (Table S3).

**Figure 1.**
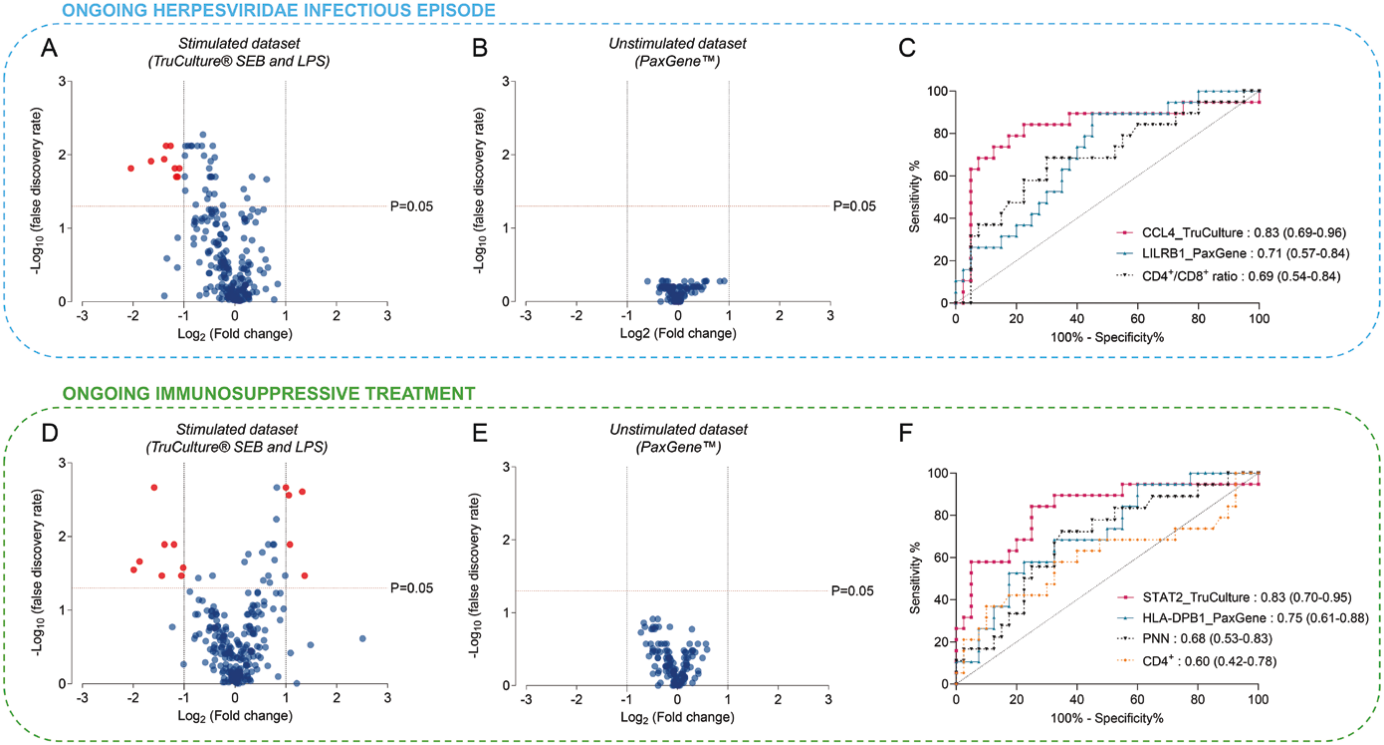
Comparison of the capacity of gene expressions obtained from stimulated and unstimulated datasets to discriminate allo-HSCT patients according to ongoing Herpesviridae infectious episode or immunosuppressive treatment. Volcano plots of gene expression after SEB or LPS stimulation (A and D) or unstimulated (B and E) differing between allo-HSCT patients with or without ongoing Herpesviridae infectious episode (A and B) or ongoing immunosuppressive treatment (D and E). Fold change (*x*-axis) is plotted against statistical significance (y axis for each gene). Red circles represent differentially expressed genes. ROC curves obtained from the comparison between allo-HSCT recipients with and without ongoing infectious episodes (*n* = 19 and *n* = 40, respectively) (C) or with and without immunosuppressive treatment (*n* = 19 and *n* = 40, respectively) (F) based on the genes with the highest AUC values in each condition (stimulated or unstimulated), as well as quantitative cell counts with the highest AUC. CCL4 and STAT2 gene expression levels were obtained following LPS and SEB stimulation, respectively. AUC [95% CI] is indicated for each parameter. *P* < .05 is considered significant. Abbreviations: CI, confidence interval; HSCT, hematopoietic stem cell transplantation; LPS, lipopolysaccharide; PNN, polynuclear neutrophils; SEB, staphylococcal enterotoxin B .

Overall, 121 genes out of the 144 genes included in the panel were commonly expressed in both the stimulated (LPS and SEB) and unstimulated datasets. Given that these 121 genes had different expression levels according to the LPS or SEB stimulation, the final 242 gene expression levels were considered for analysis. Among these, nine (3.7%) were differentially expressed between patients with or without an ongoing infectious episode (FDR correction *P* adj < 0.05; 2-FC threshold; Figure 1A, Datasource 1). Conversely, in the unstimulated dataset, no DEG was identified according to this clinical outcome (Figure 1B). The AUC of these 242 genes showed that, in the stimulated dataset, CCL4 post-LPS exhibited the highest performance for discriminating between patients with or without ongoing infectious episode (AUC of 0.83 [0.69–0.96]) compared to LILRB1 (AUC of 0.71 [0.57–0.84]) in the unstimulated dataset. Interestingly, using a large immunophenotyping panel, the highest AUC was 0.69 [0.54–0.84] for CD4^+^/CD8^+^ ratio to discriminate patients with ongoing Herpesviridae infection (Figure 1C). All AUCs calculated for the whole gene panel in the stimulated and unstimulated datasets, as well as the AUCs for the cell counts, are detailed in Datasource 1.

We then applied the same approach to evaluate whether analysis of the stimulated and unstimulated datasets could help discriminate between patients with ongoing immunosuppressive treatment and those without. In the stimulated dataset (LPS and SEB), among the 242 gene expression levels considered for analysis, 13 (5.4%) genes were differentially expressed between patients with and without immunosuppressive treatment (FDR correction *P* adj < 0.05; 2-FC threshold; Figure 1D, Datasource 1). Once again, no DEG was identified in the unstimulated dataset according to this clinical outcome (Figure 1E). The AUC of these 242 genes showed that, in the stimulated dataset, STAT2 post-SEB had the highest performance for discriminating between patients with or without ongoing immunosuppressive treatment (AUC of 0.83 [0.70–0.95]) compared to HLA-DPB1 (AUC of 0.75 [0.61–0.88]) in the unstimulated dataset. Based on the AUCs of the different cell counts, the transcriptomic approach using the stimulated dataset had a higher discriminative capacity to differentiate between patients with and without ongoing immunosuppressive treatment compared to polynuclear neutrophils, which had the highest AUC among all cell counts (AUC of 0.68 [0.53–0.83]; Figure 1F).

Using PCA for the projection of patients’ transcriptomic profiles, we then calculated the Euclidean distance of each allo-HSCT recipient to the centroid of the HV population, which served as a reference for a functional immune response. We hypothesized that the greater the Euclidean distance, the more patients had an altered immune response. As previously observed [4], the Euclidean distances were greater in patients with ongoing infectious episodes and immunosuppressive treatment (median [IQR] without infectious episode 9.71 [3.84–12.81] vs. with infectious episode 13.77 [11.47–19.25], *P* adj < 0.010 and median [IQR] without immunosuppressive treatment 9.71 [4.93–12.6] vs. with immunosuppressive treatment 13.77 [11.34-18.43], *P* adj < 0.027; Table S4). When conducting the same analyses on PaxGene samples, that is, the unstimulated dataset, the Euclidean distances were not greater according to the presence of ongoing infectious episode or immunosuppressive treatment (Figure S1A) nor any other parameter (Table S5). This loss of association, along with the reduced discriminative capacity of genes observed in the unstimulated dataset, appears to be explained by a reduced heterogeneity among allo-HSCT recipients, regardless of the clinical outcome, as indicated by the SD of the Euclidean distance (Figure S1B) and the inertia of the point cloud formed by the allo-HSCT immune profiles (Figure S1C), both of which were significantly lower in the unstimulated condition compared to the stimulated one (SD: 3.814 vs. 6.977 and inertia: 39.4 *vs.* 87.5, *P-*value < 0.001).

Overall, these findings suggest that, in the context of allo-HSCT, immune function assessments are more informative than unstimulated analyses in revealing immune function alterations, which could help improve the monitoring of post-transplant immune reconstitution by complementing the classical markers currently used.

### Immune function assessment, a valuable tool to obtain clinically relevant clustering in the context of sepsis

After demonstrating the relevance of immune function assessment in the context of allo-HSCT, we subsequently assessed its added value in another clinical context, that is, patients undergoing sepsis. Overall, 28 out of the 30 patients with sepsis, as well as the 10 HVs initially included in the Albert-Vega et *al*.’s study, were analyzed herein, as 2 PaxGene samples had not been collected. Patients with sepsis and HVs were matched for age (66 [60-79] years for patients with sepsis *vs.* 73 [72-75] years for HVs, *P-*value = 0.15) and sex (sex-male ratio 0.7 for patients with sepsis *vs.* 0.5 for HVs, *P-*value = 0.26). Among the 28 patients, 7 developed a hospital-acquired infection (HAI) during ICU stay, and 3 had died at day 28 (Table S6).

**Figure 2.**
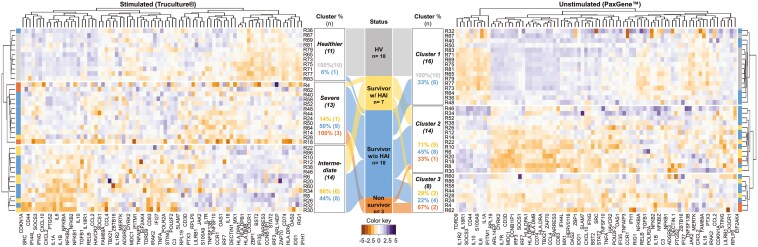
PAM clustering analysis of transcriptomic data from patients with sepsis in stimulated and unstimulated conditions. Hierarchical clustering was performed on 10 healthy volunteers (HVs) and 28 patients with sepsis. Using the PAM method with correlation distance, 3 clusters of individuals were revealed using (left) gene expression fold change in the post-SEB stimulation condition (Healthier; *n* = 11, Severe; *n* = 13, Intermediate; *n* = 14) or (right) normalized gene expression in the unstimulated condition (Cluster 1; *n* = 16, Cluster 2; *n* = 14, Cluster 3; *n* = 8). For both heatmaps, darker purple colors indicate upregulated genes while darker orange colors indicate downregulated genes. Between the two heatmaps, an alluvial plot illustrates the repartition (in percentage) of HVs (*n* = 10, gray), survivor patients with (*n* = 7, yellow) or without (*n* = 18, blue) hospital-acquired infection (HAI), and non-survivors (*n* = 3, orange) within the different clusters generated post-stimulation (left) or under unstimulated conditions (right). The percentages displayed in each box represent the proportion of the respective population included within each cluster. Abbreviations: HAI, hospital-acquired infection; HVs, healthy volunteers.

Overall, 81 genes out of the 96 genes included in the panel were commonly expressed in both the stimulated (SEB) and unstimulated datasets. Multivariate clustering analysis on the 81 gene expression levels obtained from the stimulated dataset resulted in 3 clusters with the same composition as previously described by Albert-Vega et *al.* [17] (Figure 2 left, Table S7). The first cluster (*n* = 11) grouped together all the HVs and one patient with sepsis, constituting the healthier cluster, gathering immunocompetent individuals. The second cluster (*n* = 13) included all non-survivors, hence designated as the severe cluster. It was characterized by a diminished immune responsiveness upon *in vitro* SEB stimulation and a specific modulation of genes previously described to be associated with mortality, such as MDC1 and IFI44L. The third cluster (*n* = 14) included 86% (6/7) of the patients with sepsis who developed an HAI, forming the intermediate cluster. As demonstrated by Albert-Vega et *al.*, these patients exhibited, among others, an upregulation of the HLA family and interferon-related genes, suggesting a potential for immune recovery, implying that patients identified in this cluster may benefit from immunostimulatory therapy. Of note, comparison of mHLA-DR levels, considering only clusters with a sufficient number of patients for statistically relevant analysis, showed similar levels between the intermediate and severe clusters, without statistical differences.

When applying the same analyses on the dataset obtained from PaxGene samples, an equivalent of the healthier cluster obtained with the stimulated dataset, which included all HVs, was identified. However, this cluster also included four of nine patients with sepsis who had been classified within the severe cluster identified post-SEB stimulation, including patients who exhibited an evident and profound alteration of immune function post-stimulation (Figure 2, right). This discrepancy demonstrates the loss of ability to distinguish immunocompetent individuals among a sepsis population based on gene expression analysis from unstimulated whole blood.

Altogether, these results highlight a notable discrepancy in patient stratification according to immune alteration profiles when determined with or without *in vitro* stimulation, and suggest that, during the course of sepsis, immune function assessment can reveal distinct immune profiles that are coherent with clinical characteristics.

## Discussion

In the present study, we aimed to evaluate the added value of IFA in capturing altered immune function compared to unstimulated assays in two distinct clinical contexts. To do so, we compared transcriptomic data obtained after a nonspecific whole-blood stimulation in TruCulture with those acquired using PaxGene samples, employing the same analytical pipeline.

In the context of allo-HSCT, immune cell counts such as TCD4^+^ cell count and CD4^+^/CD8^+^ ratio are classically quantified to monitor immune reconstitution post-transplant [24, 25]. However, despite their widespread clinical use, these approaches provide no information regarding the qualitative characteristics of this immune reconstitution [16, 26]. In this regard, IFAs have demonstrated complementary value to these classically used methods. Gjaerde et *al.* conducted proteomic analysis following whole-blood stimulation in TruCulture, revealing heterogeneity in cytokine production among patients and identifying a cluster with reduced responses, suggesting possible functional immune deficiency [5]. Similarly, Mouton et *al.* used nonspecific TruCulture stimulation coupled with a transcriptomic approach to capture a broad range of immune profiles, identifying altered immune function profiles associated with ongoing immunosuppressive treatment [4]. They also reported the ability of a post-stimulation gene expression analysis to discriminate patients with ongoing Herpesviridae infectious episodes. In the present study, the results obtained without *in vitro* stimulation did not reveal any DEG associated with either of these clinical outcomes. In addition, gene expression data from the unstimulated dataset had lower discriminative power than that of the stimulated dataset, which is likely due to reduced transcriptomic heterogeneity in the absence of stimulation. As a result, the variation in Euclidean distances was much lower when analyzing the unstimulated dataset compared to the stimulated dataset, preventing the observation of a possible association of the Euclidean distance from the unstimulated profiles with any clinical event or characteristic, in contrast to the associations observed for the stimulated profiles. Finally, gene expression following stimulation exhibited better discriminative power than cell counts to differentiate patients according to both clinical outcomes. The present findings are thus in line with the conclusions of Mouton et *al.* [4], which underlined that a patient stratification approach post-stimulation using transcriptomic data is more effective than solely analyzing cell counts in revealing the heterogeneity of immune profiles during post-transplant reconstitution. Importantly, the absence of stimulation does not allow to capture this heterogeneity, further supporting the interest of IFA in the allo-HSCT context, as also suggested by Naik et *al.* [16].

In the context of sepsis immune monitoring, several basal-state biomarkers (i.e. without prior stimulation) are routinely used in clinical practice, such as mHLA-DR, CD4^+^/CD8^+^ ratio, and circulating IL-10 [27]. Persistent low mHLA-DR expression [28–30], a decrease in CD4^+^/CD8^+^ ratio [31–34], as well as an increase in circulating IL-10 [35] have indeed been reported to predict mortality in septic shock. Regarding the use of IFA in this context, Antonakos *et al.* demonstrated that TNF-α production post-LPS stimulation on day 3 post-sepsis onset could discriminate patients with sepsis from healthy control subjects [36]. Additionally, Mazer et *al.* used IFN-γ and TNF-α ELISpot assays following stimulation with anti-CD3/anti-CD28 antibodies and LPS, respectively, to depict an early, profound, and sustained suppression of functional immunity in deceased patients [37]. The added value of IFA was also underlined by Albert-Vega *et al.* [17], who reported that the use of IFA enabled to identify a group of patients characterized by a potential for immune recovery, a finding that could not be observed based on mHLA-DR alone. The present study confirms that patient stratification according to post-SEB stimulation transcriptomic profiles is more effective than using the commonly employed mHLA-DR marker to underline the heterogeneity in sepsis. Moreover, the patient stratification obtained in the study by Albert-Vega *et al.* [17] was confirmed herein using the stimulated dataset but could not be replicated using the unstimulated dataset. Importantly, the clusters obtained from the unstimulated dataset appeared less clinically relevant, as they grouped together HVs with patients who were categorized in the ‘Severe’ cluster using the stimulated dataset. Altogether, the present findings reinforce the relevance of IFA employment in the sepsis setting, as also suggested by Wang *et al.* [6].

The present study has certain limitations that need to be addressed. Unfortunately, the low number of non-survivors in the sepsis cohort precluded statistical analyses for the identification of DEGs under stimulated or unstimulated conditions, as performed in the allo-HSCT cohort. Studies with larger sample sizes will be essential to fully evaluate the clinical utility of IFAs and their potential benefits for patient care. Finally, studies incorporating longitudinal follow-ups of immunocompromised patients at various stages of their conditions would be of interest.

Overall, the present analyses show that the conclusions obtained through a clustering-based stratification of post-stimulation data, in two different clinical contexts, could not be replicated using unstimulated samples. These results reinforce the interest of IFA as a complementary tool to traditional immunomonitoring methods, as already well demonstrated for specific immunity in infectious contexts, such as SARS-CoV-2 [13] and *Mycobacterium tuberculosis* [11]. The design of the present study enabled us to highlight, for the first time, the added value of the stimulation step in identifying immune function alterations. This observation could pave the way for, or at least encourage, the broader implementation of IFA as a complementary tool in immunomonitoring.

## Supplementary Material

uxaf051_suppl_Supplementary_Figures_Tables_1

uxaf051_suppl_Supplementary_Materials_1

## Data Availability

The data that support the findings of this study are available on request form the corresponding author, W.M.

## Clinical trial registration

The REALISM study protocol (NCT02638779) was approved by the regional ethics committee (*Comité de Protection des Personnes Sud-Est II*, number 2015–42-2). The ‘VaccHemInf’ study protocol (NCT03659773) was approved by the regional ethics committee (*Comité de Protection des Personnes Sud-Est V*, Grenoble, France, number 69HCL17_0769).
