# Supplementary material for: The pivotal role of immune functional assays in deciphering immune function alterations
Source: Clin Exp Immunol. 2025 Aug 9;219(1):uxaf051. doi: 10.1093/cei/uxaf051 (PMC12411760; doi:10.1093/cei/uxaf051)
Supplement: uxaf051_suppl_Supplementary_Figures_Tables_1 [file uxaf051_suppl_supplementary_figures_tables_1.zip › Figure S1.pptx]

## Slide 1
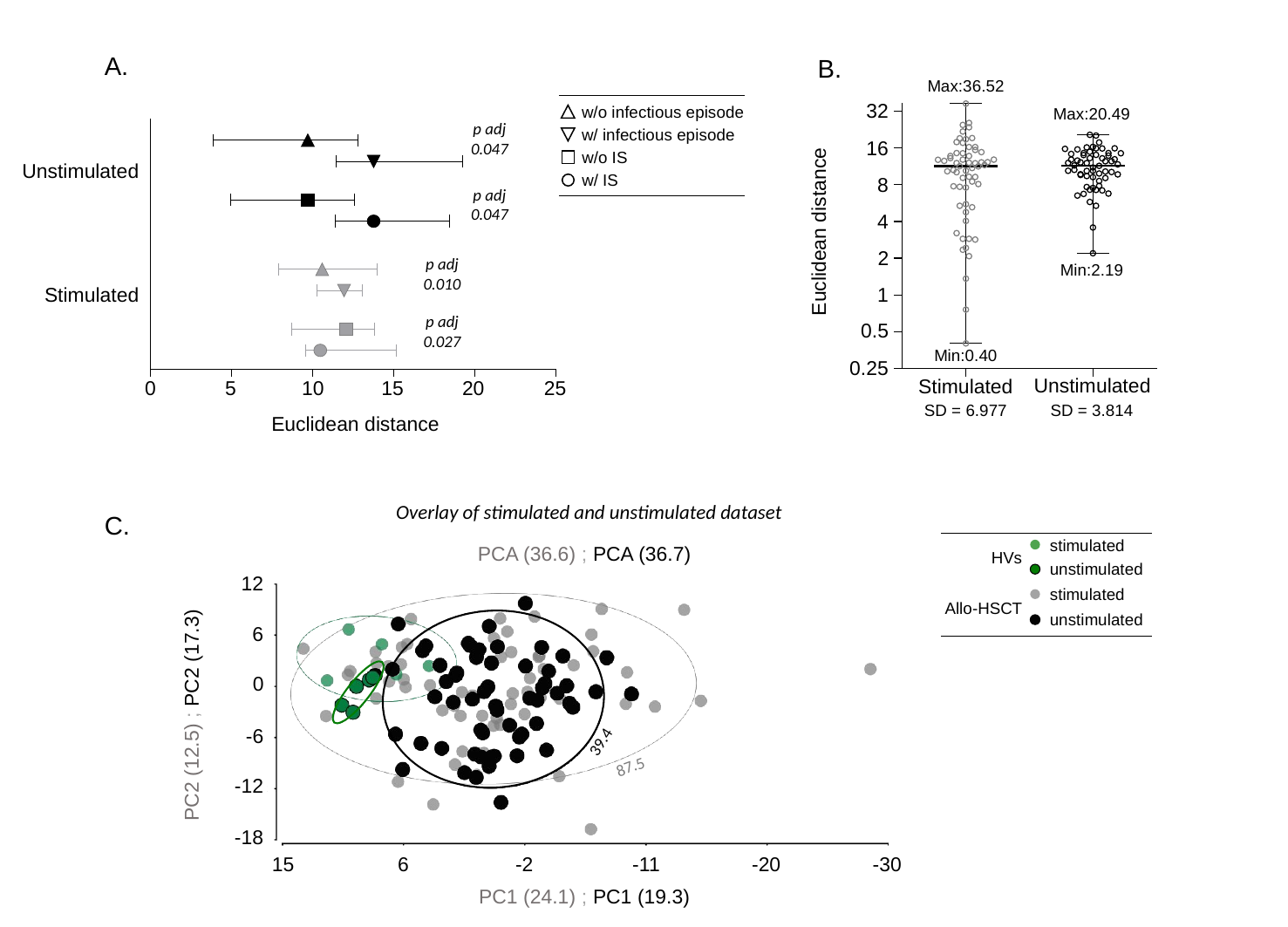

A.
B.
Max:36.52
32
Max:20.49
16
8
4
Euclidean distance
2
Min:2.19
1
0.5
Min:0.40
0.25
Unstimulated
Stimulated
SD = 6.977
SD = 3.814
w/o infectious episode
p adj
0.047
w/ infectious episode
w/o IS
Unstimulated
w/ IS
p adj
0.047
p adj
0.010
Stimulated
p adj
0.027
0
5
10
15
20
25
Euclidean distance
Overlay of stimulated and unstimulated dataset
stimulated
HVs
unstimulated
stimulated
Allo-HSCT
unstimulated
PCA (36.6) ; PCA (36.7)
12
6
0
PC2 (12.5) ; PC2 (17.3)
-6
-12
-18
15
6
-2
-11
-20
-30
PC1 (24.1) ; PC1 (19.3)
39.4
87.5
C.
