# Supplementary material for: The pivotal role of immune functional assays in deciphering immune function alterations
Source: Clin Exp Immunol. 2025 Aug 9;219(1):uxaf051. doi: 10.1093/cei/uxaf051 (PMC12411760; doi:10.1093/cei/uxaf051)
Supplement: uxaf051_suppl_Supplementary_Figures_Tables_1 [file uxaf051_suppl_supplementary_figures_tables_1.zip › Supplementary_Material_130825.docx]

Supplementary Material

# Supplementary Material and Methods

**Isolation of RNA from PaxGene™ whole-blood**

After thawing, blood RNA tubes were centrifuged at 3,000g for 10 min at room temperature (RT). The supernatant was decanted, followed by the addition of 4 ml RNase-free water and redissolving of the pellet. Next, the tube was centrifuged again at 3,000 g for 10 min at RT, and the supernatant was discarded. The pellet was resuspended in 350 µl of buffer BR1 and transferred to a microcentrifuge tube. Next, 300 µl of buffer BR2 and 40 µl of proteinase K were added, mixed, and incubated for 10 min at 55°C using a shaker-incubator at 450 rpm. This lysate was transferred to a PaxGene shredder (Qiagen, Valencia, CA) spin column and centrifuged at 20,000 g for 3 min at RT. The supernatant of the flow-through fraction was transferred to a fresh microcentrifuge tube, and 350 µl of 100% ethanol was added and mixed. Then 700 µl of this sample was transferred to a PaxGene™ RNA spin column and centrifuged at 10,000g for 1 min at RT. The flow-through was discarded, and this step was repeated with the remaining sample from the previous step.

Next, 350 µl of buffer BR3 was added onto the RNA spin column, which was centrifuged at 10,000g for 1 min at RT. The flow-through was discarded, and 10 µL of DNase 1 was mixed with 70 µl of buffer RDD in a separate tube. This was pipetted onto the spin column membrane and incubated at 20–30°C for 15 min. Then 350 µl of buffer BR3 was added onto the RNA spin column, which was centrifuged at 10,000 g for 1 min at RT. The flow-through was then discarded. Next, 500 µl of buffer BR4 was added onto the RNA spin column, which was centrifuged at 10,000 g for 1 min at RT. The flow-through was discarded, and 500 µl of buffer BR4 was added again onto the RNA spin column and centrifuged at 10,000 g for 3 min at RT. The flow-through was discarded. The RNA spin column was transferred to a 1.5-ml microcentrifuge tube, and 40 µl of buffer BR5 was pipetted onto the column to elute the membrane-bound RNA. The column was then centrifuged at 10,000 g for 1 min at RT. This step was repeated with the same 40-µl buffer BR5, and the eluate was incubated at 65°C for 5 min, then chilled on ice.

# Supplementary Tables

**Supplementary Table S1. Nanostring targeted genes and accession numbers for the allo-HSCT study, based on the Mouton et al. study** (1)**.**

| **Gene Name** | **Accession number** |
| --- | --- |
| *ADGRE3* | NM_032571.2 |
| *B2M* | NM_004048.2 |
| *BANK1* | NM_001083907 |
| *BATF* | NM_006399.3 |
| *BATF3* | NM_018664.2 |
| *BCL2* | NM_000657.2 |
| *BST2* | NM_004335.2 |
| *C1QB* | NM_000491.3 |
| *C3* | NM_000064.2 |
| *CCL23* | NM_145898.1 |
| *CCL3* | NM_002983.2 |
| *CCL4* | NM_002984.2 |
| *CCR1/ RANTES R* | NM_001295.2 |
| *CCR5* | NM_000579.1 |
| *CCR7* | NM_001838.2 |
| *CCRL2* | NM_003965.4 |
| *CD209/DC-SIGN* | NM_021155.2 |
| *CD244* | NM_016382.2 |
| *CD27 / TNFRSF7* | NM_001242.4 |
| *CD274* | NM_014143.3 |
| *CD4* | NM_000616.4 |
| *CD40* | NM_001250.4 |
| *CD44* | NM_001001392.1 |
| *CD45RA* | NM_002838.4 |
| *CD79A* | NM_001783.3 |
| *CD79B* | NM_021602.2 |
| *CD80* | NM_005191.3 |
| *CD83* | NM_004233.3 |
| *CDKN1A* | NM_000389.2 |
| *CEBPB* | NM_005194.2 |
| *CSF1* | NM_000757.4 |
| *CSF2* | NM_000758.2 |
| *CSF2RB* | NM_000395.2 |
| *CX3CR1* | NM_001337.3 |
| *CXCL10/IP10* | NM_001565.1 |
| *CXCL11* | NM_005409.4 |
| *CXCL2/MIP2alpha* | NM_002089.3 |
| *CXCL9* | NM_002416.1 |
| *CXCR1* | NM_000634.2 |
| ***DECR1*** | **NM_001359.1** |
| *FCGR2B* | NM_001002273.1 |
| *FCRL5* | NM_031281 |
| *FCRLA* | NM_032738 |
| *FOXP3* | NM_014009.3 |
| *GBP1* | NM_002053.1 |
| *GBP5* | NM_052942.3 |
| *GZMB* | NM_004131.3 |
| *HAVCR2* | NM_032782.3 |
| *HLA-DMB* | NM_002118.3 |
| *HLA-DPA1* | NM_033554.2 |
| *HLA-DPB1* | NM_002121.4 |
| *HLA-DRA* | NM_019111.3 |
| ***HPRT1*** | **NM_000194.1** |
| *ICAM1* | NM_000201.2 |
| *IDO1* | NM_002164.3 |
| *IFIH1/MDA5* | NM_022168.2 |
| *IFIT2* | NM_001547.4 |
| *IFITM1* | NM_003641.3 |
| *IFNG* | NM_000619.2 |
| *IGHG1* | NM_001040077 |
| *IL1B* | NM_000576.2 |
| *IL1RN* | NM_000577.3 |
| *IL27* | NM_145659.3 |
| *IL2RA* | NM_000417.1 |
| *IL8* | NM_000584.2 |
| *IL9* | NM_000590.1 |
| *IRAK2* | NM_001570.3 |
| *IRF4* | NM_002460.1 |
| *IRF5* | NM_002200.3 |
| *IRF7* | NM_001572.3 |
| *JAK2* | NM_004972.2 |
| *LAG3* | NM_002286.5 |
| *LAMP3* | NM_014398.3 |
| *LIF* | NM_002309.3 |
| *LILRA3* | NM_006865.3 |
| *LILRA5* | NM_181879.2 |
| *LILRB1* | NM_032571.2 |
| *LILRB4* | NM_012276.3 |
| *LTA* | NM_000595.2 |
| *MME* | NM_000902.2 |
| *MS4A1 / CD20* | NM_152866.2 |
| *MX1* | NM_002462.2 |
| *NFKB1* | NM_003998.2 |
| *NFKB2* | NM_002502.2 |
| *NFKBIA* | NM_020529.1 |
| *NFKBIZ* | NM_001005474.1 |
| *PDCD1LG2* | NM_025239.3 |
| *PECAM1* | NM_000442.3 |
| *PML* | NM_002675.3 |
| ***POLR2A*** | **NM_000937.2** |
| *POU2AF1* | NM_006235 |
| *POU2F2* | NM_002698.2 |
| *PPIB* | NM_000942.4 |
| *PTX3* | NM_002852.3 |
| *RARRES3* | NM_004585.3 |
| *RELB* | NM_006509.2 |
| *RPL19* | NM_000981.3 |
| *SERPING1* | NM_000062.2 |
| *SLAMF1* | NM_003037.2 |
| *SLAMF7* | NM_021181.3 |
| *SOCS1* | NM_003745.1 |
| *SOCS3* | NM_003955.3 |
| *SPP1* | NM_000582.2 |
| *SRC* | NM_005417.3 |
| *STAT1* | NM_007315.2 |
| *STAT2* | NM_005419.2 |
| *TAGAP* | NM_054114.3 |
| *TAP1* | NM_000593.5 |
| *TAP2* | NM_000544.3 |
| *TBP* | NM_001172085.1 |
| *TCF7* | NM_003202.2 |
| *TLR7* | NM_016562.3 |
| *TNFA* | NM_000594.2 |
| *TNFAIP3* | NM_006290.2 |
| *TNFAIP6* | NM_007115.2 |
| *TNFRSF17/BCMA* | NM_001192.2 |
| *TNFRSF4* | NM_003327.2 |
| *TNFRSF9* | NM_001561.4 |
| *TNFSF10* | NM_003810.2 |
| *TNFSF13B* | NM_006573.4 |
| *TNFSF15* | NM_001204344.1 |
| *TRAF1* | NM_005658.3 |
| *XCL1* | NM_002995.1 |
| *ZAP70* | NM_001079.3 |

Housekeeping genes used for the gene expression analysis are depicted in bold

**Supplementary Table S2. Nanostring targeted genes and accession numbers for the sepsis study, based on the Albert-Vega et al. study** (2)**.**

| Target genes | Accession number |
| --- | --- |
| *ADGRE3* | NM_032571.2 |
| *ARL14EP* | NM_152316.1 |
| *BST2* | NM_004335.2 |
| *C3* | NM_000064.2 |
| *CCL2* | NM_002982.3 |
| *CCL4* | NM_002984.2 |
| *CCNB1IP1* | NM_182849.2 |
| *CCR1* | NM_001295.2 |
| *CD3D* | NM_000732.4 |
| *CD44* | NM_001001392.1 |
| *CD74* | NM_001025159.1 |
| *CD83* | NM_004233.3 |
| *CDKN1A* | NM_000389.2 |
| *CLEC7A/DECTIN1* | NM_197954.2 |
| *CX3CR1* | NM_001337.3 |
| *CXCL10/IP10* | NM_001565.1 |
| *CXCL2/MIP2A* | NM_002089.3 |
| ***DECR1*** | **NM_001359.1** |
| *DDX58/RIG1* | NM_014314.3 |
| *DYRK2* | NM_003583.3 |
| *EIF2AK4* | NM_001013703.2 |
| *FAM89A* | NM_198552.2 |
| *HAVCR2/TIM3* | NM_032782.3 |
| *HLA-DMB* | NM_002118.3 |
| *HLA-DPA1* | NM_033554.2 |
| *HLA-DPB1* | NM_002121.4 |
| *HLA-DRA* | NM_019111.3 |
| ***HPRT1*** | **NM_000194.1** |
| *IDO1* | NM_002164.3 |
| *IFI27* | NM_005532.3 |
| *IFI35* | NM_005533.3 |
| *IFI44L* | NM_006820.2 |
| *IFIH1* | NM_022168.2 |
| *IFITM1* | NM_003641.3 |
| *IFNG* | NM_000619.2 |
| *IL10* | NM_000572.2 |
| *IL18* | NM_001562.2 |
| *IL18R1* | NM_003855.2 |
| *IL1A* | NM_000575.3 |
| *IL1B* | NM_000576.2 |
| *IL1R2* | NM_004633.3 |
| *IL6* | NM_000600.1 |
| *IL7R* | NM_002185.2 |
| *IRAK2* | NM_001570.3 |
| *IRF3* | NM_001571.5 |
| *IRF7* | NM_001572.3 |
| *JAK2* | NM_004972.2 |
| *LILRB1* | NM_001081637.1 |
| *MDC1* | NM_014641.2 |
| *MERTK* | NM_006343.2 |
| *MX1* | NM_002462.2 |
| *NFKB1* | NM_003998.2 |
| *NFKB2* | NM_002502.2 |
| *NFKBIA* | NM_020529.1 |
| *NFKBIZ* | NM_001005474.1 |
| *OAS1* | NM_001032409.1 |
| *OAS2* | NM_016817.2 |
| *POLR2A* | NM_000937.2 |
| *POU2F2* | NM_002698.2 |
| *PTGS2* | NM_000963.1 |
| *PTX3* | NM_002852.3 |
| *RARRES3* | NM_004585.3 |
| *RELB* | NM_006509.2 |
| *RPL19* | NM_000981.3 |
| *RPLP0* | NM_001002.3 |
| *S100A9* | NM_002965.2 |
| *SLAMF7* | NM_021181.3 |
| *SOCS1* | NM_003745.1 |
| *SOCS3* | NM_003955.3 |
| *SRC* | NM_005417.3 |
| *STAT2* | NM_005419.2 |
| *TBX21* | NM_013351.1 |
| ***TBP*** | **NM_001172085.1** |
| *TDRD9* | NM_153046.2 |
| *TGFB1* | NM_000660.3 |
| *TMEM173/STING* | NM_198282.1 |
| *TNFA* | NM_000594.2 |
| *TNFAIP3* | NM_006290.2 |
| *TNFSF10* | NM_003810.2 |
| *TNFSF13B* | NM_006573.4 |
| *ZAP70* | NM_001079.3 |
| *ZBP1* | NM_001160419.2 |
| *ZBTB16* | NM_006006.4 |
| *121601901-HERV0116* | chr12:112972627-112975754 |

Housekeeping genes used for the gene expression analysis are depicted in bold

**Supplementary Table S3. Clinical data in allo-HSCT recipients**

|  | **Allo-HSCT recipients (n=59)** | **Healthy Volunteers**  **(n=5)** | **p.adj**  **(Wilcoxon)** |
| --- | --- | --- | --- |
| **Demographics** |  |  |  |
| Age, median [IQR] | 44 [33.5-60.5] | 53 [49-61] | 0.21 |
| Male, n (%) | 34 (57.6) | 3 (60) | >0.99 |
| **Time from transplantation, months (median [IQR])** | 6.5 [5.8-8.3] | NA | NA |
| **Hematological and transplant-related characteristics, n (%)** |  |  |  |
| *Underlying hematological disease* |  |  |  |
| Acute myeloid leukemia and related neoplasms | 31 (52.5) | NA | NA |
| Myelodysplastic syndromes | 7 (11.9) | NA | NA |
| Myeloproliferative neoplasms | 1 (1.7) | NA | NA |
| B-lymphoblastic leukemia/lymphoma | 11 (18.6) | NA | NA |
| T-lymphoblastic leukemia/lymphoma | 2 (3.4) | NA | NA |
| Mature neoplasms: T, NK, or B cells | 3 (5.1) | NA | NA |
| Hodgkin lymphoma | 1 (1.7) | NA | NA |
| Others | 3 (5.1) | NA | NA |
| *CR before the engraftment* | 53 (89.8) | NA | NA |
| *Donor types* |  |  |  |
| Geno-identical | 18 (30.5) | NA | NA |
| Haplo-identical | 11 (18.6) | NA | NA |
| Pheno-identical | 29 (49.2) | NA | NA |
| Fully matched | 23 (79.3) | NA | NA |
| HLA mismatched | 6 (20.7) | NA | NA |
| *Stem cell source* |  |  |  |
| Peripheral blood cells | 47 (79.7) | NA | NA |
| Bone marrow | 11 (18.6) | NA | NA |
| Cord blood | 1 (1.7) | NA | NA |
| *Conditioning regimen* |  |  |  |
| MAC | 37 (62.7) | NA | NA |
| RIC | 22 (37.3) | NA | NA |
| TBI | 18 (30.5) | NA | NA |
| ATG | 34 (57.6) | NA | NA |
| **GvHD, n (%)** |  |  |  |
| *GvHD prophylaxis* |  |  |  |
| ATG | 34 (57.6) | NA | NA |
| Calcineurin inhibitors | 58 (98.3) | NA | NA |
| Mycophenolate Mofetil | 32 (54.2) | NA | NA |
| Corticosteroids | 0 (0) | NA | NA |
| Methotrexate | 14 (23.7) | NA | NA |
| Post-transplant cyclophosphamide | 22 (37.3) | NA | NA |
| *History of GvHD between transplantation and inclusion* |  |  |  |
| Acute GvHD | 42 (71.2) | NA | NA |
| Grade I/II/III | 28/12/2 | NA | NA |
| Chronic GvHD | 8 (13.6) | NA | NA |
| Grade I/II/III | 3/3/2 | NA | NA |
| *GvHD status at inclusion* |  |  |  |
| No history of GvHD | 15 (25.4) | NA | NA |
| Resolved GvHD | 23 (39.0) | NA | NA |
| Active GvHD (acute or chronic) | 21 (35.6) | NA | NA |
| **Immunophenotyping, median [Q1-Q3]** |  |  |  |
| Lymphocytes (NV, 1000-2800/µL) | 1210 [790-2220] | NA | NA |
| CD3^+^ T-lymphocytes (NV, 521-1772/µL) | 666 [422.5-1017] | NA | NA |
| CD3^+^ CD4^+^ T-lymphocytes (NV, 336-1126/µL) | 261 [124-345] | NA | NA |
| Naïve CD4^+^ (CD45^+^CCR7^+^) (NV, 121-456/µL) | 16.0 [8.0-33.5] | NA | NA |
| Central memory CD4^+^ (CD45RA^-^CCR7^+^) (NV, 92-341/µL) | 54.5 [21.5-85.8] | NA | NA |
| Effector memory CD4^+^ (CD45RA^-^CCR7^-^) (NV, 59-321/µL) | 129 [87.0-207.8] | NA | NA |
| Differentiated memory CD4^+^ (CD45RA^+^CCR7^-^) (NV, 11-102/µL) | 4.5 [0.0-29.75] | NA | NA |
| CD3^+^ CD8^+^ T-lymphocytes (NV, 125-780/µL) | 366 [223-618] | NA | NA |
| Naïve CD8^+^ (CD45^+^CCR7^+^) (NV, 86-257µL) | 20.0 [10.0-52.0] | NA | NA |
| Central memory CD8^+^ (CD45RA^-^CCR7^+^) (NV, 19-93/µL) | 10.0 [3.3-17.0] | NA | NA |
| Effector memory CD8^+^ (CD45RA^-^CCR7^-^) (NV, 15-162/µL) | 157 [75-281] | NA | NA |
| Differentiated memory CD8^+^ (CD45RA^+^CCR7^-^) (NV, 39-212/µL) | 156 [57.8-309.3] | NA | NA |
| CD4^+^/CD8^+^ ratio (NV, 0.9-6) | 0.57 [0.35-0.98] | NA | NA |
| CD20^+^ B-lymphocytes (NV, 64-593/µL) | 173 [90-379] | NA | NA |
| Immunoglobulin G titers (NV, 7-16 g/L) | 7.5 (6.0-9.9) | NA | NA |
| **Post-transplant immunomodulatory therapy at inclusion, n (%)** |  |  |  |
| IS therapy at inclusion# | 19 (32.2) | NA | NA |
| IVIG infusion(s) | 40 (67.8) | NA | NA |
| Time since last IVIG infusion (days), median [IQR] | 128 [89.8-174.5] | NA | NA |
| DLI | 6 (10) | NA | NA |

#Immunosuppressive therapies included ciclosporin (n = 9), tacrolimus (n = 4), corticosteroids (n = 8), and ruxolitinib (n = 3)

*Abbreviations: ATG*, antithymocyte globulin; *CM*, central memory; *CR*, complete remission; *D*, donor; *DM*, differentiated memory; *DLI*, donor lymphocyte infusion; *EM*, effector memory; *GvHD*, graft versus host disease; *IS*, immunosuppressive; *IVIG*, intravenous immunoglobulins; *MAC*, myeloablative conditioning; NK, natural killer; *NV*, normal values; *R*, recipient; *RIC*, reduced-intensity conditioning; *TBI,* total body irradiation.

**Supplementary Table S4. Association between transplant-related factors and Euclidean distance calculated from the HV population centroid to each allo-HSCT recipient projected on the PCA, based on the stimulated TruCulture results obtained from the restricted post-stimulation dataset.**

| **Clinical variable** | **No Euclidean distance median [Q1-Q3] Allo-HSCT** | **Yes Euclidean distance median [Q1-Q3] Allo-HSCT** | ***p.adj (Wilcoxon)*** |
| --- | --- | --- | --- |
| **Ongoing immunosuppressive treatment** | **9.71 [4.93-12.6] n= 40** | **13.77 [11.34-18.43] n= 19** | **0.027** |
| **Herpesviridae infectious episode^#^** | **9.71 [3.84-12.81**  **n=40** | **13.77 [11.47-19.25]**  **n=19** | **0.010** |
| Recipients > 60 years old | 11.8 [8.32-14.48] n= 44 | 8.11 [3.63-14.53] n= 15 | 0.933 |
| Sex - Male | 11.59 [5.55-14.79] n= 25 | 11.34 [7.63-13.77] n= 34 | 0.933 |
| Acute myeloid leukemia | 111.55 [4.93-16.66] n= 28 | 11.36 [7.64-12.82] n= 31 | 0.933 |
| Complete remission* | 17.86 [11.36-18.89]  n= 5 | 11.32 [5.38-13.77] n= 53 | 0.299 |
| Reduced intensity conditioning | 11.59 [5.38-14.79] n= 37 | 11.34 [8.15-14.29] n= 22 | 0.933 |
| Radiotherapy | 11.32 [7.7-14.46] n= 41 | 12.69 [5.81-14.35] n= 18 | 0.933 |
| Anti-thymocyte globulin treatment | 12.22 [3.21-13.77] n= 25 | 11.15 [8.66-14.71] n= 34 | 0.933 |
| Peripheral blood cells | 10.76 [5.34-13.28] n= 12 | 11.36 [7.64-14.63] n= 47 | 0.933 |
| Haploidentical donor | 11.34 [7.76-13.95] n= 48 | 12.52 [4.71-18.37] n= 11 | 0.933 |
| History of acute GvHD  since transplant | 12.1 [7.7-13.77] n= 17 | 11.15 [5.42-14.52] n= 42 | 0.933 |
| History of chronic GvHD  since transplant | 11.59 [5.46-14.67] n= 51 | 11.34 [10.13-12.06] n= 8 | 0.933 |
| T cells CD4+ > 200 cells/µL | 11.79 [6.19-14.29] n= 22 | 10.97 [7.58-14.54] n= 37 | 0.933 |
| T cells CD4+/CD8+ ratio >1 | 12 [7.47-14.95] n= 44 | 9.27 [6.18-12.51] n= 15 | 0.933 |
| CMV reactivation status | 10.48 [5.3-12.86]  n= 46 | 16.26 [11.59-18.89]  n= 13 | 0.948 |

*Data were missing for one patient

^#^Detection of Herpesviridae reactivation within 2 weeks of inclusion

*Abbreviations: GvHD*, graft versus host disease

**Supplementary Table S5. Association between transplant-related factors and Euclidean distance calculated from the HV population centroid to each allo-HSCT recipient projected on the PCA, based on the unstimulated PaxGene results.**

| **Clinical variable** | **No Euclidean distance median [Q1-Q3] Allo-HSCT** | **Yes Euclidean distance median [Q1-Q3] Allo-HSCT** | ***p.adj (Wilcoxon)*** |
| --- | --- | --- | --- |
| Ongoing immunosuppressive treatment | 12.05 [8.69-13.81] n= 40 | 10.48 [9.58-15.19] n= 19 | 0.957 |
| Herpesviridae infectious episode^#^ | 10.59 [7.88-13.96]  n=40 | 11.95 [10.29-13.05]  n=19 | 0.957 |
| Recipients > 60 years old | 11.83 [8.91-13.92] n= 44 | 11.6 [9.19-13.89] n= 15 | 0.957 |
| Sex - Male | 11.6 [9-13.22] 25 | 11.78 [8.87-14.43] n= 34 | 0.957 |
| Acute myeloid leukemia | 11.63 [9.39-14.38] n= 28 | 11.79 [8.83-13.68] n= 31 | 0.957 |
| Complete remission* | 13.22 [11.92-13.83] n= 5 | 10.62 [8.56-13.92] n= 53 | 0.957 |
| Reduced intensity conditioning | 12.15 [10.18-13.92] n= 37 | 9.81 [7.35-13.14] n= 22 | 0.957 |
| Radiotherapy | 11.88 [9.53-13.85] n= 41 | 10.07 [7.32-14.4] n= 18 | 0.957 |
| Anti-thymocyte globulin treatment | 12.06 [9-14.68] n= 25 | 11.26 [8.87-13.75] n= 34 | 0.957 |
| Peripheral blood cells | 12.32 [8.56-14.38] n= 12 | 11.6 [9.09-13.84] n= 47 | 0.957 |
| Haploidentical donor | 11.69 [9.31-13.99] n= 48 | 11.67 [8.28-12.9] n= 11 | 0.957 |
| History of acute GvHD  since transplant | 11.88 [7.96-13.92] n= 17 | 11.63 [9.13-13.75] n= 42 | 0.957 |
| History of chronic GvHD  since transplant | 10.93 [8.6-13.84] n= 51 | 12.57 [11.29-14.3] n= 8 | 0.957 |
| T cells CD4+ > 200 cells/µL | 11.87 [9.71-14.43] n= 22 | 11.67 [7.96-13.5] n= 37 | 0.957 |
| T cells CD4+/CD8+ ratio >1 | 11.83 [9.4-13.87] n= 44 | 10.18 [7.62-14.09] n= 15 | 0.957 |
| CMV reactivation status | 10.78 [7.77-13.85]  n= 46 | 11.95 [10.65-14.12]  n= 13 | 0.957 |

*Data were missing for one patient

^#^Detection of Herpesviridae reactivation within 2 weeks of inclusion

*Abbreviations: GvHD*, graft versus host disease

**Supplementary Table S6. Clinical data for patients with sepsis**

|  | **Patients with sepsis (n=28)** | | **Healthy Volunteers (n=10)** | | **p.value**  **(wilcoxon)** | |  |
| --- | --- | --- | --- | --- | --- | --- | --- |
| **Admission data** | |  | |  | |  | |
| Sex, male, n (%) | | 20 (71.4) | | 5 (50) | | 0.15 | |
| Median age, years [IQR] | | 66.00 [60.00-78.25] | | 73 [72-75] | | 0.26 | |
| Median BMI, kg/m2 [IQR] | | 24.61 [21.79-26.58] | | NA | | NA | |
| Median SAPS II [IQR] | | 47.00 [39.50-55.25] | | NA | | NA | |
| Median SOFA score (day 1) [IQR] | | 9.00 [7.75-10.00] | | NA | | NA | |
| Mechanical ventilation, n (%) | | 19 (67.9) | | NA | | NA | |
| Median plasma lactate level, mM [IQR] | | 2.20 [1.70-2.80] | | NA | | NA | |
| Shock, n (%) | | 19 (67.9) | | NA | | NA | |
| Median CCI [IQR] | | 2.00 [0.75-4.00] | | NA | | NA | |
| *Comorbidities^a^, n (%)* | |  | | NA | | NA | |
| 0 | | 7 (25.0) | | NA | | NA | |
| ≥ 1 | | 21 (75.0) | | NA | | NA | |
| *Primary site of infection, n (%)* | |  | | NA | | NA | |
| Abdominal | | 11 (39.3) | | NA | | NA | |
| UTI | | 2 (7.1) | | NA | | NA | |
| SST | | 2 (7.1) | | NA | | NA | |
| Pulmonary | | 10 (35.7) | | NA | | NA | |
| Others | | 3 (10.8) | | NA | | NA | |
| *Type of primary infection, n (%)* | |  | | NA | | NA | |
| Community-acquired | | 21 (75.0) | | NA | | NA | |
| Hospital-acquired | | 7 (25.0) | | NA | | NA | |
| *Documentation of infection, n (%)* | |  | | NA | | NA | |
| Gram-negative | | 5 (17.9) | | NA | | NA | |
| Gram-positive | | 10 (35.7) | | NA | | NA | |
| Virus | | 0 | | NA | | NA | |
| Fungal | | 4 (14.3) | | NA | | NA | |
| Co-infection | | 3 (10.7) | | NA | | NA | |
| Non-documented infection | | 6 (21.4) | | NA | | NA | |
| Hydrocortisone, n (%) | | 7 (25.0) | | NA | | NA | |
| **Day 3–4 data** | |  | |  | |  | |
| *Immunology* | |  | |  | |  | |
| Median mHLA-DR, Ab/C [IQR] | | 4511.32 [3125.37-8053.97] | | NA | | NA | |
| Median TNFα secretion post-LPS stimulation, pg/mL [IQR] | | 1089.78 [669.71-1733.68] | | NA | | NA | |
| **Outcomes** | |  | | NA | | NA | |
| Vasopressor requirement, n (%) | | 26 (92.9) | | NA | | NA | |
| Median vasopressor duration, days [IQR] | | 1.91 [1.04-3.34] | | NA | | NA | |
| Hospital-acquired infection, n (%) | | 7 (25.0) | | NA | | NA | |
| Median ICU length of stay, days [IQR] | | 9.00 [6.50-13.00] | | NA | | NA | |
| Missing data | | 1 | | NA | | NA | |
| Median hospital length of stay, days [IQR] | | 28.00 [13.25-43.50] | | NA | | NA | |
| Missing data | | 3 | | NA | | NA | |
| Mortality at day 28, n (%) | | 3 (10.7) | | NA | | NA | |

SAPS II was calculated after admission and SOFA score was measured after 24 h of ICU stay.

^a^: Presence of comorbidities was affirmative when at least one of the following comorbidity was present in the patient: chronic pulmonary disease, heart failure, myocardial infarction, ulcer, diabetes, renal failure, or malignant solid tumor.

*Abbreviations*: *BMI*, body mass index; *SAPS II*, simplified acute physiology score; *SOFA*, sequential organ failure assessment; *CCI*, Charlson comorbidity index; *UTI*, urinary tract infection; *SST*, skin and soft tissue; *HLA-DR*, human leukocyte antigen DR; *TNFα*, tumor necrosis factor alpha; *LPS*, lipopolysaccharide; *ICU*, intensive care unit

**Supplementary Table S7. Comparison of the individual composition of the clusters obtained from stimulated TruCulture analysis before and after removal of patients and genes from the original published sepsis cohort** (2)**.**

|  | | **TruCulture restricted dataset** | | |
| --- | --- | --- | --- | --- |
| **TruCulture published data** |  | **Cluster 1** | **Cluster 2** | **Cluster 3** |
|  | **Cluster 1** |  |  | R81; R83; R65; R67; R69; R71; R73; R75; R77; R79; R36 |
|  | **Cluster 2** | R32; R10; R12; R34; R38; R46; R8; R60; R20; R22; R26; R30; R6; R86 |  |  |
|  | **Cluster 3** |  | R14; R18; R24; R28; R4; R40; R44; R48; R50; R52; R58; R62; R64 |  |

HV are depicted in blue, patients with sepsis in black, non-survivors in red, and hospital-acquired infections in orange. There was no difference in the clustering between the two datasets.

**Supplementary Table S8. Bivariate analyses between clusters 1, 2, and 3 upon SEB stimulation or unstimulated and clinical and biological parameters.**

|  | Stimulated dataset (TruCulture®) | | | | Unstimulated dataset (PaxGene™) | | | |
| --- | --- | --- | --- | --- | --- | --- | --- | --- |
|  | **Healthy Cluster** | **Intermediate Cluster** | **Severe Cluster** | ***P* value** | **Cluster 1** | **Cluster 2** | **Cluster 3** | ***P* value** |
| **Status** |  |  |  | 0.0025 |  |  |  | 0. 0025 |
| Healthy, n (%) | 10 (90.91) | 0 (0) | 0 (0) |  | 10 (62.5) | 0 (0) | 0 (0) |  |
| Patient with sepsis, n (%) | 1 (9.09) | 14 (100) | 13 (100) |  | 6 (37.5) | 14 (100) | 8 (100) |  |
| Comorbidities*^a^, n (%) | 1 (9.09) | 11 (78.57) | 9 (69.23) | 1.00 | 4 (25) | 11 (78.57) | 6 (75) | 1.00 |
| **Day 3-4** |  |  |  |  |  |  |  |  |
| Median mHLA-DR (Ab/C)*, median [IQR] | 2974.94 [2974.94-2974.94] | 4342.29 [2909.57-7849.43] | 4705.34 [3342.76-7969.29] | 1.00 | 4332.78 [3025.08-6714.53] | 4414.33 [3541.38-7794.51] | 4523.74 [2484.27-8417.49] | 1.00 |
| **Outcomes** |  |  |  |  |  |  |  |  |
| Hospital-acquired infections*, n (%) | 0 (0) | 6 (42.86) | 1 (7.69) | 0.5597 | 0 (0) | 5 (35.71) | 2 (25) | 1.00 |
| Mortality at day 28*, n (%) | 0 (0) | 0 (0) | 3 (23.08) | 0.9020 | 0 (0) | 1 (7.14) | 2 (25) | 1.00 |

Categorical variables were analyzed using the Chi-squared test, and numerical variables using the t-test or Wilcoxon test, as appropriate.

*Parameters measured exclusively for septic patients. ^a^ Presence of comorbidities was affirmative when at

least one of the following comorbidity was present in the patient: chronic pulmonary disease, heart failure,

myocardial infarction, ulcer, diabetes, renal failure, or malign solid tumour.

*Abbreviations*: *HLA-DR* human leukocyte antigen DR

# Supplementary Figure

Figure S1. Comparison of the individual transcriptomic profiles of allo-HSCT recipients in stimulated and unstimulated conditions.

(A) Forest plot representing the median [IQR] Euclidean distance from allo-HSCT recipients to the centroid of the HV population, calculated from the PCA under stimulated (light grey) or unstimulated (dark grey) conditions. (B) Comparison of the dispersion of Euclidean distance from allo-HSCT recipients to the centroid of the HV population, calculated from the PCA under stimulated (light grey) or unstimulated (dark grey) conditions. (C) Overlay of the 2 principal component analyses (PCA) of gene expression derived from post-LPS and –SEB stimulated (light colors) and unstimulated (dark colors) data from HVs (n=5, green circles) and allo-HSCT recipients (n=59, grey circles). Each individual transcriptomic profile, represented by a circle, was projected onto the 2 first principal components (PC). Values annotated on the ellipses represent the inertia of the associated point cloud. Comparison between the median [IQR] Euclidean distance from patients with or without infectious episode or ongoing immunosuppressive treatment was performed using non-parametric unpaired Wilcoxon test with Benjamini correction. p.adj <0.05 is considered as significant.

*Abbreviations: HVs,* healthy volunteers*; HSCT,* hematopoietic stem cell transplantation; *IS,* immunosuppressive treatment; *LPS,* lipopolysaccharide*; PC,* principal component*; PCA,* principal component analysis*; SD,* standard deviation*; SEB,* staphylococcal enterotoxin B*.*

# Supplementary Reference

1. Mouton W, Conrad A, Alcazer V, Boccard M, Bodinier M, Oriol G, Subtil F, Labussière-Wallet H, Ducastelle-Lepretre S, Barraco F, et al. Distinct Immune Reconstitution Profiles Captured by Immune Functional Assays at 6 Months Post Allogeneic Hematopoietic Stem Cell Transplantation. *Transplantation and Cellular Therapy* (2023) 29: doi: 10.1016/j.jtct.2022.10.025

2. Albert Vega C, Oriol G, Bartolo F, Lopez J, Pachot A, Rimmelé T, Venet F, Leray V, Monneret G, Delwarde B, et al. Deciphering heterogeneity of septic shock patients using immune functional assays: a proof of concept study. *Sci Rep* (2020) 10: doi: 10.1038/s41598-020-73014-2
